# Detection of Premalignant Cervical Lesions via *Maackia amurensis* Lectin-Based Biosensors

**DOI:** 10.3390/bios16010024

**Published:** 2025-12-29

**Authors:** Ricardo Zamudio Cañas, Verónica Vallejo Ruiz, María Eugenia Jaramillo Flores, Raúl Jacobo Delgado Macuil, Valentín López Gayou

**Affiliations:** 1 Laboratorio de Bionanotecnología, Centro de Investigación en Biotecnología Aplicada, Instituto Politécnico Nacional (IPN-CIBA), Tepetitla 90700, Mexico; rzamudioc1700@alumno.ipn.mx (R.Z.C.); rdelgadom@ipn.mx (R.J.D.M.); 2 Laboratorio de Biología Molecular, Centro de Investigación Biomédica de Oriente, Instituto Mexicano del Seguro Social, Metepec 74360, Mexico; veronica_vallejo@yahoo.com; 3 Laboratorio de Biopolímeros, Escuela Nacional de Ciencias Biológicas, Instituto Politécnico Nacional (IPN-ENCB), Ciudad de Mexico 07738, Mexico; mjarfl@ipn.mx

**Keywords:** lectin-based biosensor, Fourier transform infrared (FTIR) spectroscopy, premalignant lesions, cervical cancer (CC), chemometric analysis

## Abstract

Early detection of premalignant cervical lesions is essential for improving cervical cancer outcomes; however, current screening methods frequently lack adequate sensitivity and specificity. This research introduces a diagnostic platform that integrates lectin-based biosensors with spectral and multivariate analysis. The biosensors are composed of gold nanoparticles (AuNPs) conjugated with *Maackia amurensis* (MAA) lectin, which selectively binds to α2,3-linked sialic acid. Validation was performed using cervical cancer cell lines (SiHa, HeLa, C33A), fibroblasts, and cervical scrapes, and specificity was verified by enzymatic removal of sialic acids. Spectral data were obtained using attenuated total reflectance Fourier transform infrared spectroscopy (ATR-FTIR) and analyzed by principal component analysis (PCA). Application of PCA to the 1600–1350 cm^−1^ spectral region, using 99% confidence ellipses, enabled clear differentiation between samples negative and positive for intraepithelial lesions in a double-blind study of 58 patients. The MAA biosensors exhibited high sensitivity and specificity, comparable to established diagnostic methods. These results indicate that the combination of ATR-FTIR spectroscopy, MAA lectin-based biosensors, and chemometric analysis provides a robust and reliable approach for early detection of premalignant cervical lesions, with considerable potential to enhance patient outcomes.

## 1. Introduction

According to global statistics. In 2022, cervical cancer (CC) accounted for an estimated 662,301 new cases and 348,784 deaths globally, with the majority occurring in low- and middle-income countries [1,2]. This high mortality rate underscores the urgent need for improved screening and detection methods. Traditional screening methods, including Papanicolaou (Pap) smears and human papillomavirus (HPV) testing, are widely used but often lack the sensitivity and specificity needed to detect premalignant lesions early. Pap smears typically demonstrate a sensitivity of 55–80% and a specificity of 85–90% [3,4]. HPV testing offers approximately 90% increased sensitivity, though this often results in reduced specificity, and it is conditioned to the infection with HPV [5]. These findings highlight the necessity for more reliable and accurate diagnostic tools [6].

A defining feature of many cancers, including cervical cancer, is the overexpression of sialic acids, particularly α2,3 and α2,6-linked sialic acid conformations, on glycoproteins and glycolipids [7,8,9]. Sialic acid, a terminal sugar residue, plays a critical role in cancer biology by mediating cell signaling, adhesion, and metastasis. In 2010, López-Morales et al. demonstrated through histochemical analysis that sialic acid expression progressively increases with cervical cancer progression [10]. As a result, targeting specific sialic acid conformations, particularly α2,3-linked sialic acid, is a promising strategy for early cancer detection. Recent advances in biosensor technology have facilitated the development of innovative diagnostic methods [11]. Optical biosensors employing gold nanoparticles (AuNPs) as transducers and conjugated to proteins as biological recognition elements have exhibited high specificity for analyte binding, enabling the detection of a broad spectrum of diseases [12,13,14,15]. AuNPs are especially valuable in oncology for imaging and diagnostic applications due to their localized surface plasmon resonance (LSPR), biocompatibility, and ease of functionalization [16,17,18]. For instance, Huang et al. demonstrated that a colorimetric AuNPs method for detecting PAX1 methylation in cervical scrapes provides high sensitivity for cervical cancer screening [19], and more recently, Navarro Chica et al. developed a nanoparticle-based colorimetric assay for rapid, early screening of high-risk HPV variants 16 and 18 [20]. These studies underscore the versatility of AuNPs for functionalization with diverse biomolecules. This study proposes the conjugation of *Maackia amurensis* (MAA) lectin, which exhibits strong affinity for α2,3-linked sialic acids [21], to AuNPs as a targeted approach for the early detection of cervical lesions. The effectiveness of these biosensors is enhanced by the surface-enhanced infrared absorption (SEIRA) effect, which exploits the electromagnetic fields generated by gold nanoparticles. This amplification allows the detection of trace molecules, such as sialic acid, by enhancing their vibrational modes near the metallic surface, thereby substantially increasing the sensitivity and detection capabilities of optical biosensors [11,22,23]. Additionally, chemometric techniques such as principal component analysis (PCA) and confidence ellipses are essential in biomedical research for interpreting complex spectral data and further improving diagnostic accuracy and reliability [24,25,26]. By combining chemometric analysis with biosensor technology, we can develop robust diagnostic tools capable of distinguishing between negative and positive samples for Intraepithelial lesions.

Herein, we propose and validate a novel method for detecting premalignant cervical lesions using lectin-based optical biosensors that target the potential biomarker α2,3-linked sialic acid. The interaction between MAA biosensors and cervical scrape samples was analyzed by ATR-FTIR spectroscopy combined with chemometric analysis (PCA) to evaluate the method’s effectiveness and potential clinical utility. To our knowledge, no similar approach has been previously described. This strategy is intended to complement existing screening methods and enhance clinical applicability.

## 2. Materials and Methods

### 2.1. Materials

All reagents used for gold nanoparticle synthesis, including HEPES, glacial acetic acid, and *Maackia amurensis* (MAA) lectin, were obtained from Sigma-Aldrich (St. Louis, MO, USA). Chloroauric acid (HAuCl_4_) and medium-molecular-weight chitosan (poly(D-glucosamine), 75–85% deacetylation) were utilized in the synthesis process. Glacial acetic acid was diluted to a 1% solution with deionized water before use. All aqueous solutions were prepared using deionized water.

### 2.2. Construction of MAA Biosensors

AuNPs were prepared following the methodology proposed by Zamudio et al., 2024 [27], with a modification regarding lectin conjugation. A 400 µL AuNPs solution was placed in a microtube with 5 µg of MAA lectin to promote their interaction for 24 h at 14 °C. The mixture was then centrifuged at 8000 rpm for 30 min, and the supernatant was removed. The obtained pellet was resuspended in 400 µL 1 mM HEPES. The Lectin-based biosensor was characterized by UV-Vis, ATR-FTIR, Transmission electron microscopy (TEM) and dynamic light scattering (DLS) using a Genesys 40 Visible Spectrophotometer (Thermo Scientific, Waltham, MA, USA), Bruker 70v FT-IR vacuum spectrometer equipped with an A225/Q Platinum ATR module and a 2.4 single-reflection diamond crystal (Bruker Optics, Ettlingen, Germany), Zetasizer Nano S-90 (Malvern Instruments, Worcestershire, UK) and a JEM2200 (EOL Ltd., Tokyo, Japan) at the Centro de Nanociencias y Micro y Nanotecnologías (Mexico City, México), respectively.

### 2.3. Cell Lines and Cell Culture Conditions

The SiHa, HeLa, and C33A human cervical cancer cell lines were acquired from the Centro de Investigación Biomédica de Occidente (CIBO) in Guadalajara, Mexico, with acknowledgment to Dr. Adriana Aguilar Lemarroy of the CIBO Immunology Division cell bank. Primary fibroblasts were sourced from the Escuela Nacional de Ciencias Biológicas (ENCB-IPN) in Mexico City. The uniformity of all cell cultures was verified using RT-qPCR, following the methodology of Jagadeeshaprasad et al., 2022 [28]. Cervical cancer cell lines were maintained in Dulbecco’s Modified Eagle Medium (DMEM; Gibco, Billings, MT, USA) supplemented with 10% fetal bovine serum (FBS; Gibco, USA), whereas fibroblasts were cultured in DMEM containing 15% FBS and 1% penicillin/streptomycin. Cultures were incubated at 37 °C in a humidified environment with 5% CO_2_. Once cells reached 80% confluence, they were passaged using 1× trypsin supplemented with 0.25% EDTA.

### 2.4. Cell-MAA Biosensor Interactions

Prior to spectroscopic measurements, all cell lines were detached using 1× trypsin-EDTA (0.25%) for 5 min, then centrifuged at 2500 rpm for 5 min. After discarding the supernatant, cells were washed once with PBS buffer and centrifuged again at 2500 rpm for 5 min. The resulting cell pellets were resuspended in distilled water. Cervical scrapes were initially stored in methanol. Before interacting with MAA biosensors, all samples were washed with PBS buffer and centrifuged at 2500 rpm for 5 min. For each experiment, 40,000 cells from either cell lines or cervical scrapes were used. The cell suspension was placed in a microtube, and 50 µL of assembled MAA biosensors was added, allowing direct interaction for 1 h at room temperature. The mixture was then centrifuged at 2500 rpm for 5 min; after removing the supernatant, the cell + MAA biosensor pellets were resuspended in 50 µL of 1× PBS. All samples were processed in the same manner to ensure valid comparisons.

### 2.5. Neuraminidase Bioassay

To remove the sialic acid on the cell surface, the neuraminidase enzyme from *Clostridium perfringens* was purchased from Sigma-Aldrich, as it catalyzes the cleavage of the terminal N-acetylneuraminic acid (Neu5Ac) from glycoconjugates [29]. Cells in suspension were obtained from the SiHa cell line and fibroblasts. Cell counting was performed using a Neubauer chamber, and cell viability was assessed using Trypan blue. Subsequently, the desired cell concentration was taken. Washes were performed with PBS, followed by centrifugation at 2500 rpm for 5 min, and resuspension in 10 mM HEPES buffer. 0.5 units of neuraminidase enzyme from *Clostridium perfringens* were added per 100,000 cells, and the reaction was maintained under slight agitation for 30 min at 37 °C. Finally, centrifugation was performed at 2000 rpm for 5 min, and the cells were resuspended in 1 mM HEPES buffer prior to interaction with MAA biosensors.

### 2.6. Sample Collection

Cervical scrapes were collected at the National Health Centre, Manuel Avila Camacho, from the Mexican Institute of Social Security (IMSS) in Puebla City, from April 2022 to February 2023. Patients included in the study were diagnosed with low- or high-grade squamous intraepithelial lesions by a pathologist. All the women included in the study signed a consent form before sample collection. A total of 58 cervical scrape samples were collected, including 28 with normal cytology and 30 from patients diagnosed with low- and high-grade squamous intraepithelial lesions.

### 2.7. Spectral Analysis

For each assay, 2 µL of sample solution was applied to the ATR crystal and dried at room temperature to minimize water interference. The crystal was thoroughly cleaned with distilled water before each use and between samples. Spectral data were collected in the mid-infrared range (4000–400 cm^−1^), averaging 132 scans per spectrum at a resolution of 4 cm^−1^. To ensure sample viability, measurements for cervical cancer cell lines were performed in triplicate over three days, resulting in a total of nine spectra per cell type. For cervical scrapes, only a single measurement was obtained per sample due to limited cell count.

### 2.8. Data Analysis

Principal Component Analysis (PCA) was used as an unsupervised method to cluster and differentiate samples based on vibrational data obtained by ATR-FTIR (Attenuated Total Reflectance Fourier Transform Infrared spectroscopy). The analysis considered both the full mid-infrared range (4000–400 cm^−1^) and specific spectral regions. PCA reduces data dimensionality while retaining most of the variability, facilitating visualization and interpretation of complex datasets like biological spectra. To ensure comparability, all spectra were preprocessed using 9-point smoothing, baseline correction, and min/max normalization with OPUS v7.0 software. Standardization ensures that each variable contributes equally to the PCA and prevents dominance by variables with larger scales. Confidence ellipses representing a 99% confidence interval were used to indicate the certainty of sample classification within groups, visually demonstrating group variability and overlap to assess the significance of clustering. Data preprocessing and analysis were performed with OPUS v7.0 and Unscrambler X v10.4, while result plotting was conducted using ORIGIN Pro 2025b (Learning Edition).

For statistical analysis, a One-Way ANOVA with the Tukey test was performed in OriginPro 2025b (Learning Edition) to determine statistical significance; *p*-values ≤ 0.05 were considered significant. GraphPad Prism 8 was used for plotting the results.

## 3. Results

### 3.1. Characterization of Maackia amurensis Lectin-Based Biosensors

Although various methods are available for synthesizing gold nanoparticles (AuNPs), the use of chitosan in a green synthesis approach provides distinct advantages. Chitosan functions as both a reducing and stabilizing agent, as well as an inherent functionalizer of AuNPs, simplifying downstream conjugation processes [30,31,32]. During synthesis, chitosan undergoes hydrolysis and fragmentation, producing shorter polymer chains that serve as primary reducing agents during the multi-step reduction of Au (III) ions. The resulting –NH_3_^+^ groups on chitosan enhance colloidal stability, maintaining well-dispersed AuNPs [27,33,34,35]. Furthermore, the abundant hydroxyl and amine groups on chitosan enable efficient biomolecule attachment. This method produces amine-functionalized AuNPs that will be attached to carboxyl groups of the MAA lectin, facilitating effective biosensor assembly.

**Figure 1:**
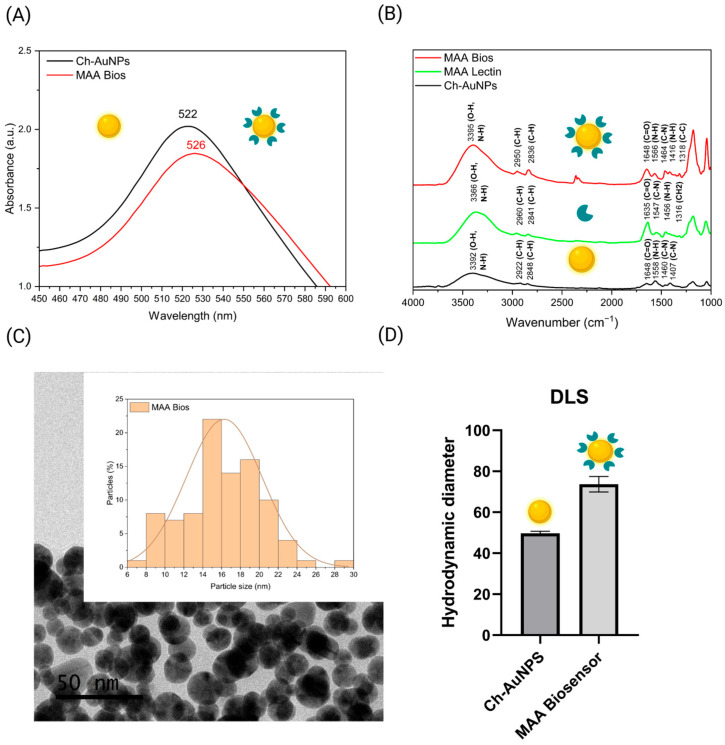
(**A**) UV-vis spectra of Ch-AuNPs (black) and conjugated with MAA lectin (red). (**B**) ATR-FTIR spectra of Ch-AuNPs (black), MAA lectin control (green), and MAA biosensors (red). (**C**) Representative TEM images of MAA biosensors with the corresponding particle size distribution histogram. (**D**) Hydrodynamic size measurements of Ch-AuNPs and MAA biosensors. Created with Biorender.com.

The conjugation of *Maackia amurensis* (MAA) lectin onto gold nanoparticles was first evidenced by a shift in the UV-vis absorbance maximum from 522 to 526 nm (Figure 1A), indicative of localized surface plasmon resonance (LSPR) effects. This red shift aligns with an increased local refractive index surrounding the nanoparticle, thereby confirming effective lectin attachment, which is characteristic of label-free surface plasmon resonance (SPR) biosensor technology [36]. Attenuated total reflectance Fourier-transform infrared (ATR-FTIR) spectroscopy provided additional insight into the surface chemistry. Chitosan-coated gold nanoparticles (Ch-AuNPs) exhibited distinctive absorption bands at 3392, 2922, 2848, 1648, 1558, 1460, and 1407 cm^−1^ (Figure 1B), which are attributable to functional groups in chitosan: O–H/N–H stretching (3392 cm^−1^), C–H stretching (2922, 2848 cm^−1^), C=O stretching (1648 cm^−1^), and amide II C–N/N–H bending (1558 cm^−1^) [30,31]. Following conjugation with MAA, new absorption features appeared at 1648, 1566, 1464, and 1318 cm^−1^, closely matching those of pure MAA lectin (1635, 1547, 1456, and 1316 cm^−1^) and indicating the presence of amide I, II, and III vibration [37]. These spectral changes, relative to both Ch-AuNPs and unbound lectin, support the occurrence of successful biomolecular interactions at the nanoparticle surface. The absence of major spectral shifts after conjugation indicates that the secondary structure and functional integrity of the lectin are preserved. Transmission electron microscopy (TEM) analysis demonstrated that the MAA biosensors retained a regular, polydisperse morphology with an average particle diameter of approximately 16 nm (Figure 1C). Dynamic light scattering (DLS) was subsequently used to further confirm conjugation. Following MAA attachment, the hydrodynamic diameter increased from 50 nm for Ch-AuNPs to 73 nm for MAA biosensors (Figure 1D), consistent with previous literature [36,38]. The observed difference between TEM and DLS measurements arises because DLS accounts for the core size and contributions from surface coatings and solvation layers, thereby reflecting changes in particle surface chemistry after functionalization.

### 3.2. Detection of Sialic Acid Terminations in Cervical Cancer Cell Lines Using ATR-FTIR

**Figure 2:**
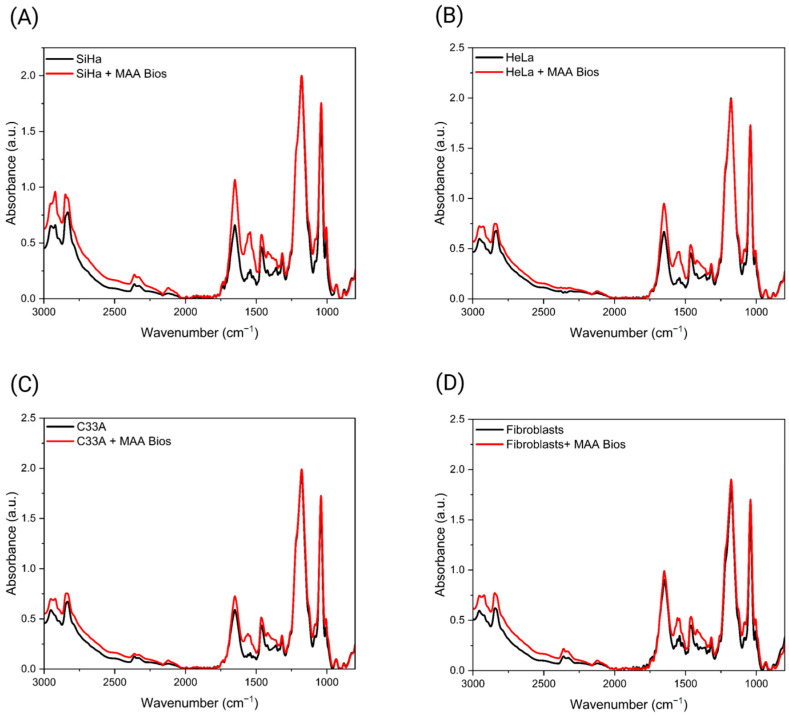
Mean ATR-FTIR spectra (baseline-corrected and min/max-normalized) of control cells (black) and cells after interaction with MAA biosensors (red) for: (**A**) SiHa, (**B**) HeLa, (**C**) C33A, and (**D**) primary fibroblasts. Created with Biorender.com.

**Figure 3:**
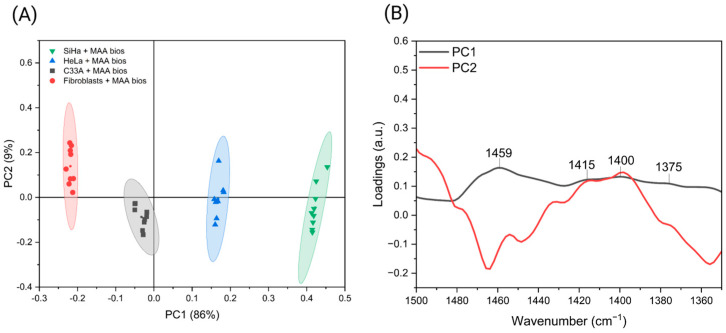
(**A**) PCA score plot with 95% confidence ellipses based on the 1500–1350 cm^−1^ spectral range, illustrating differentiation between cervical cancer cell lines and primary fibroblasts. (**B**) Corresponding loading plot for the first two principal components, highlighting the spectral variables that contribute most significantly to the observed group separation. Created with Biorender.com.

After assembly of optical MAA biosensors, these interacted with three cervical cancer cell lines (SiHa, HeLa, and C33A) and primary fibroblasts to detect sialic acid terminations on the cell surface. The interaction was analyzed by ATR-FTIR, and a signal enhancement was observed in the spectra of all biosensor-interacting samples compared to those from cells alone (Figure 2A–D). This phenomenon can be attributed to the SEIRA effect, in which the vibrational modes of α2,3-linked sialic acid are enhanced due to their contact with MAA biosensors and the electromagnetic field from AuNPs. Notably, a similar phenomenon was observed in a previous study employing *Sambucus nigra* lectin to target α2,6-linked sialic acid [27]. These enhancements were more noticeable in the range of 1600–1300 cm^−1^. Within this range, we found that the band pattern was maintained through all the samples, with characteristic bands at ∼1650, 1540, 1460, 1420, and 1315 cm^−1^. These bands are related to the C=O stretching vibration of amide I (1700–1600 cm^−1^), N-H bending vibration related to amide II (1600–1500 cm^−1^), CH_3_ bending modes (1450–1400 cm^−1^), and C-H and C-O-H related to amide III bands [5,39,40]. Several PCA models were developed using different spectral ranges to optimize differentiation. The model employing the 1500–1350 cm^−1^ range, combined with 95% confidence ellipses for PC1 and PC2, which together accounted for 95% of the total variance, effectively distinguished cervical cancer cell lines from primary fibroblasts (Figure 3A). Notably, the samples were distributed and clustered along PC1. The HPV positive SiHa and HeLa cell lines are located on the positive axis of PC1, while primary fibroblasts and the HPV-negative C33A cell line are located on the negative axis of PC1. Studies have shown that SiHa and HeLa cells have a higher expression of sialic acid; this could be attributed, among other factors, to the significantly enhanced expression of certain transcripts, such as V1 from the *ST3GAL4* gene, in these cervical carcinoma cell lines compared to non-tumorigenic cell lines. In contrast, the C33A cell line does not show this increase, which could explain why these samples are located on the negative axis of PC1 and PC2 (Figure 3A) [10,41,42]. Furthermore, the lack of overlap among the ellipses indicates that the FTIR spectra for each detection were statistically different. The corresponding loading plots identified four key bands in this region that contributed to sample discrimination: 1459, 1415, 1400, and 1375 cm^−1^ (Figure 3B). These bands might represent:

C-H bending, N-H, and C-N Amide II bands (1459 cm^−1^): Possibly reflects the interaction of MAA lectin with sialic acid on the cell surface.

Carboxylate Groups (1415 and 1400 cm^−1^): Reflects the presence of sialic acid and its interaction with the biosensor.

C-H, O-H bending (1375 cm^−1^): Could represent changes in the local environment of the sialic acid or other biomolecules upon binding.

The positive and negative charges on the amine and carboxylic acid groups of sialic acid have been reported to contribute to the binding of molecules such as serotonin [43]. In this case, the carboxyl groups of sialic acid may interact with positively charged amine residues in the MAA lectin. To optimize this interaction, slight conformational changes in both the MAA lectin and the sialic acid may ensure a snug fit. Additionally, the carboxyl groups (–COOH) of sialic acid might form hydrogen bonds with the lectin.

The bands at 1459, 1415, 1400, and 1375 cm^−1^ may indicate the presence of sialic acid and its interaction with the MAA biosensors.

### 3.3. Specificity Confirmation of MAA Biosensors via Neuraminidase Bioassay

To confirm the specificity of the MAA biosensors and to verify the spectral region associated with sialic acid and its modifications, sialic acid was enzymatically cleaved from the cell surface. Neuraminidase (Neura) from *Clostridium perfringens*, which hydrolyzes sialic acid termini from glycoconjugates without affecting the backbone, was utilized [29]. The assay was performed on the SiHa cell line and primary fibroblasts, since more variation in their sialic acid concentrations was observed in our previous analysis.

**Figure 4:**
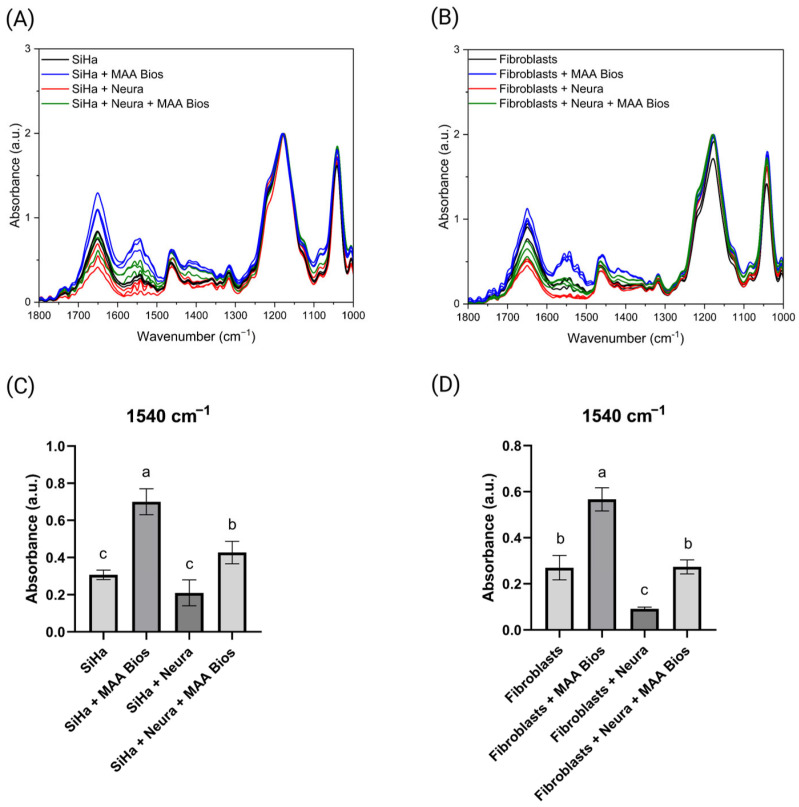
(**A**) FTIR spectra obtained from the neuraminidase bioassay on the SiHa cell line. (**B**) FTIR spectra obtained from the neuraminidase bioassay on primary fibroblasts. (**C**) Statistical analysis of absorbance at 1540 cm^−1^ in the SiHa cell line using ANOVA followed by Tukey’s post hoc test (*p* < 0.05). (**D**) Statistical analysis of absorbance at 1540 cm^-1^ in primary fibroblasts using ANOVA with Tukey’s post hoc test (*p* < 0.05). Created with Biorender.com.

A decrease in signal intensity was detected in both cell types following treatment with the Neura enzyme, particularly within the 1700–1350 cm^−1^ spectral range (Figure 4A,B). The reduction in primary fibroblast samples was more pronounced than in the SiHa cell line within the 1700–1500 cm^−1^ region (Figure 4B), where the 1650 and 1530 cm^−1^ bands associated with sialic acid are located [43,44]. This observation is consistent with higher sialic acid expression on tumor cell surfaces compared to non-tumor cells [10,45]. Consequently, spectral changes are more significant in cells with lower sialic acid concentrations after enzymatic treatment. The enzyme is reported to remove only a portion of the sialic acid [46]. In untreated cells, an increase in signal intensity was observed, consistent with previous findings across different cell lines and attributed to the surface-enhanced infrared absorption (SEIRA) effect. An increase in intensity was also observed in samples treated with the Neura enzyme and exposed to MAA biosensors, in both fibroblasts and the SiHa cell line, with some variation. The intensity values of the 1540 cm^−1^ band, associated with amide II, were analyzed using ANOVA followed by Tukey’s post hoc test (*p* < 0.05) to assess significant differences between samples (Figure 4C,D). This band was selected for its association with sialic acid and its pronounced differences across conditions [44]. For the SiHa cell line, significant differences in intensity were identified in the “SiHa + Neura + MAA Bios” samples compared to the controls “SiHa” and “SiHa + Neura” (Figure 4C). This outcome is attributed to the high concentration of sialic acid in these cells and its incomplete enzymatic cleavage. Post-treatment, sufficient sialic acid remains to generate a SEIRA effect that distinguishes these samples, although the effect is less pronounced than in the “SiHa + MAA Bios” samples, where the full biomarker concentration is present. In primary fibroblasts, a significant difference was observed only between the “Fibroblasts + Neura” samples, but not with the “Fibroblasts” controls (Figure 4D). This finding suggests that after treatment, the residual sialic acid on the cell surface is minimal, resulting in limited signal amplification compared to the “Fibroblasts + MAA Bios” samples (Figure 4D).

**Figure 5:**
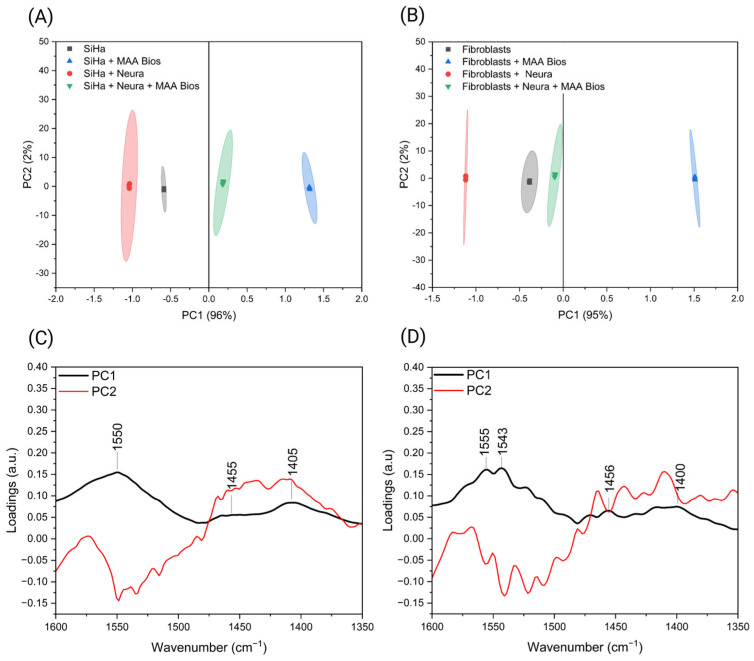
PCA score plots with 95% confidence ellipses within the 1600–1350 cm^−1^ range of the neuraminidase bioassay using (**A**) SiHa cell line and (**B**) primary fibroblasts. Loading plots derived from the PCA results of the neuraminidase bioassay using (**C**) the SiHa cell line and (**D**) primary fibroblasts. Created with Biorender.com.

Principal component analysis (PCA) was conducted in the 1600–1350 cm^−1^ region, yielding an explained variance greater than 90% for both sample types. Confidence ellipses with a 95% prediction value were applied, effectively differentiating the samples. In the SiHa cell line, the “SiHa” and “SiHa + Neura” samples were positioned on the negative axis of PC1, while samples interacting with MAA biosensors clustered on the positive axis (Figure 5A). In the fibroblast assay, only the “Fibroblasts + MAA Bios” samples clustered on the positive axis of PC1 (Figure 5B). These distinctions are attributed to the initial sialic acid concentrations in each sample type. Loading plots indicate that the bands at 1550, 1455, and 1405 cm^−1^ for the SiHa cell line (Figure 5C) and 1555, 1456, and 1400 cm^−1^ for fibroblasts (Figure 5D) are the primary contributors to sample separation, confirming their association with sialic acid and its modifications. These findings are consistent with previous classifications of cervical cancer cell lines and primary fibroblasts, which showed bands at approximately 1450 and 1400 cm^−1^ [27]. This evidence demonstrates that both the MAA biosensors and the analytical method are sensitive to variations in sialic acid concentration.

### 3.4. Differentiation of Premalignant Cervical Lesions Using MAA Biosensors and PCA

**Figure 6:**
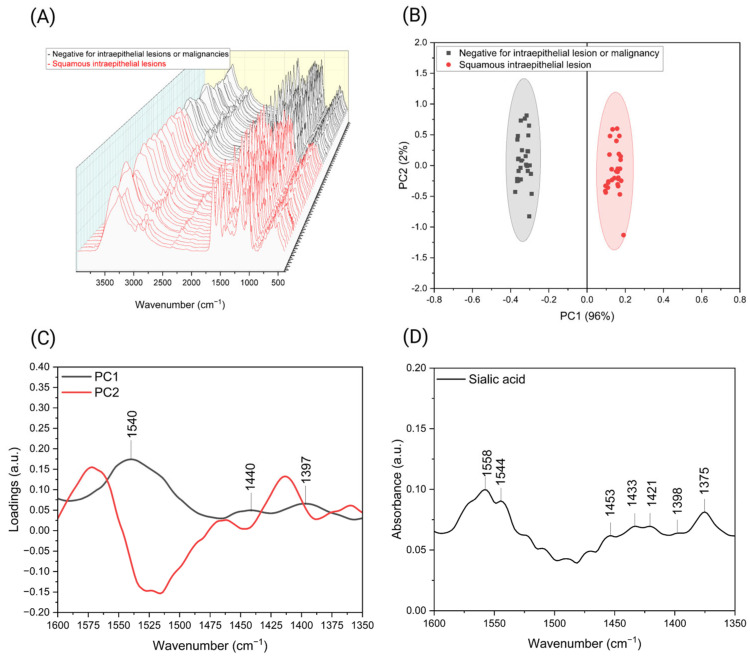
(**A**) Baseline-corrected and min/max-normalized ATR-FTIR spectra of cervical scrapes after detection with MAA biosensors. (**B**) PCA score plot with 95% confidence ellipse, based on the 1600–1350 cm^−1^ spectral range, illustrating differentiation among cervical scrape samples. (**C**) Corresponding loading plot for the first two principal components, highlighting the spectral variables within the 1600–1350 cm^−1^ region that contribute most significantly to sample discrimination. (**D**) ATR-FTIR spectra of pure sialic acid. Created with Biorender.com.

Following confirmation that the developed biosensors and methodology effectively differentiate cervical cancer (CC) cell lines from fibroblasts and demonstrate specificity to sialic acid concentration, as determined by the neuraminidase bioassay, a total of 58 spectra corresponding to the interaction of MAA biosensors with cervical scrapes were obtained. The spectra underwent nine-point smoothing, baseline correction, and normalization to reduce noise and account for sampling-related differences due to collection on different dates [24]. This preprocessing was performed using the OPUS v7.0 software installed on the FTIR equipment. From ATR-FTIR results, we observed that all samples exhibit the same band distribution in two regions: 3000–2800 cm^−1^ and 1800–900 cm^−1^ (Figure 6A).

Based on these results, PCA models were constructed for various spectral regions, with particular focus on the 1700–1300 cm^−1^ range, previously identified as being associated with sialic acid. Notably, the PCA model using the 1600–1350 cm^−1^ region, together with a 99% confidence ellipse, achieved clear separation between the two sample classes. The resulting model explains 98% of the total variance, and the distinct separation of the ellipses highlights the effectiveness of PCA in distinguishing negative and positive samples for squamous Intraepithelial lesions (Figure 6B). The loading plot (Figure 6C) revealed that bands at 1540, 1440, and 1397 cm^−1^ are responsible for the classification and sample separation. These bands also enabled differentiation in the neuraminidase (Neura) bioassay. Interestingly, samples positioned on the positive axis of PC1 corresponded to premalignant lesions, consistent with a higher biomarker concentration. This trend was also observed in the Neura bioassay, where samples with elevated sialic acid concentrations clustered on the positive axis. Furthermore, analysis of pure sialic acid spectra showed bands at ∼1544, ∼1453, and ∼1398 cm^−1^ (Figure 6D), aligning with those identified in the loading plot (Figure 6C); slight variations can be attributed to interactions with MAA biosensors. After classification, the results were compared with clinical diagnoses, including cytology, HPV DNA testing, colposcopy, and biopsy (the gold standard), with complete concordance observed. This comparison demonstrates the high accuracy of our classification method. However, to enable predictive analysis and further validate the approach, a larger sample size and the application of predictive algorithms such as LDA and SVM are required.

Finally, a comparison was made between the bands obtained from our pure sialic acid analysis, the bands reported by Nallala et al., 2020 [44], Rana et al., 2022 [47], and those derived from the loading plots for each assay. We observed slight differences in band positions. However, we identified a consistent pattern of three bands near those observed in the pure sialic acid spectrum: 1558, 1433, and 1396 cm^−1^ (Table 1). Specifically, bands at ∼1550 cm^−1^ and ∼1540 cm^−1^ are associated with amide II (C-N and N-H vibrations) [48]. These bands correspond to the N-acetyl group of sialic acid, since sialic acid has five hydroxyl groups, one N-acetyl group, and one carboxyl group [8,43,47]. On the other hand, bands at ∼1450 cm^−1^, present in all cases, are related to amide II, C-H bending, and are also reported to be part of the sialic acid backbone [46]. These results provide evidence of the interaction between the MAA lectin and sialic acid on the cell surface.

Bands at these approximate positions predominantly contribute to PC1 and allow us to differentiate between classes in all bioassays. These bands confirm their association with the sialic acid biomarker and their concentration modifications. The differences in spectral band positions can be attributed to the sample’s local microenvironment, with Cervical scrapes showing higher concentrations and greater diversity of macromolecules than cell lines.

## 4. Conclusions

In this study, we developed and validated optical biosensors based on *Maackia amurensis* (MAA) lectin and gold nanoparticles (AuNPs) for the early detection of premalignant cervical lesions. In a double-blind study using cervical scrapes from 58 patients, our biosensors targeting α2,3-linked sialic acid, analyzed by ATR-FTIR spectroscopy and chemometric methods, achieved clear differentiation between samples negative and positive for intraepithelial lesions in the 1600–1350 cm^−1^ spectral range. Principal component analysis (PCA) and loading plots identified spectral bands at approximately 1540, 1440, and 1397 cm^−1^, associated with α2,3-linked sialic acid, as the main contributors to this discrimination. This method demonstrated high sensitivity and specificity, comparable to those of conventional screening methods.

These findings suggest that integrating vibrational spectroscopy, MAA-based biosensors, and advanced chemometric analysis offers a robust and reliable approach for early detection of premalignant cervical lesions. Further research with larger and more diverse sample sets, as well as the application of supervised analysis methods such as LDA or SVM, will be important to validate and extend these results. Ultimately, this approach holds significant potential to improve early diagnosis and patient outcomes in cervical cancer screening.

## 5. Patents

The technology described in this article is subject to a patent application currently under examination by the Instituto Mexicano de la Propiedad Industrial (IMPI); Application number: MX/a/2024/015592.

## Figures and Tables

**Table 1:** Key spectral bands contributing to cell differentiation in bioassays using MAA biosensors, as determined from loading plot analysis, with comparison to pure sialic acid signals previously described by Nallala et al. (2020) [44] and Rana et al. (2022) [47].

Samples	Key Spectral Bands (cm^−1^)					
Sialic acid—Nallala [44]		1530	1438			1376
Sialic acid—Rana [47]		1528	1431			1374
Sialic Acid	1558	1544	1433	1421	1396	1375
Cell culture differentiation			1459	1415	1400	
Neuraminidase bioassay	1553		1455		1402	
Cervical Scrapes bioassay		1540	1440		1397	

## Data Availability

Data will be made available on request.
